# Experimental Evaluation of Under Slab Mats (USMs) Made from End-of-Life Tires for Ballastless Tram Track Applications

**DOI:** 10.3390/ma17215388

**Published:** 2024-11-04

**Authors:** Cezary Kraśkiewicz, Piotr Majnert, Anna Al Sabouni-Zawadzka, Przemysław Mossakowski, Marcin Zarzycki

**Affiliations:** 1 Faculty of Civil Engineering, Warsaw University of Technology, Al. Armii Ludowej 16, 00-637 Warsaw, Poland; anna.zawadzka@pw.edu.pl (A.A.S.-Z.); przemyslaw.mossakowski@pw.edu.pl (P.M.); 2 CONE AMG Sp. z o.o., ul. Zasobna 49/lok 9, 04-862 Warsaw, Poland; pmajnert@cone.com.pl (P.M.); mzarzycki@cone.com.pl (M.Z.)

**Keywords:** under slab mat, vibration isolation, floating slab system, sustainable tramway construction, recycled rubber

## Abstract

The growing population of urban areas results in the need to deal with the noise pollution from the transportation system. This study presents experimental test results of static and dynamic elastic characteristics of under slab mats (USMs) according to the procedure of DIN 45673-7. Prototype USMs based on recycled elastomeric materials, i.e., SBR granules and fibres produced from waste car tires, are analysed. Vibration isolation mats with different thicknesses (10, 15, 20, 25, 30, and 40 mm), densities (500 and 600 kg/m^3^), and different degrees of space filling (no holes, medium holes, large holes) are considered. Moreover, a practical application of the laboratory test results of USMs in the design of ballastless track structures of two different types (with a concrete slab and longitudinal beams) is presented. Deflections of the rail and the floating slab system, as well as stresses acting on the mat, are determined according to EN 16432-2. The use of shredded rubber from recycled car tires as a material component of sustainable and environmentally friendly tram track structures may be one of the most effective ways to manage rubber waste within the current trend toward a circular economy, and this study intends to introduce methods for experimental identification and analytical selection of basic static and dynamic parameters of prototype USMs.

## 1. Introduction

The ever-increasing population of major cities is putting a significant strain on the transportation system, resulting in increasingly frequent and burdensome traffic congestion. One way to improve the efficiency of cities’ transportation systems is to reduce individual transportation in favour of thoughtful public transportation that is attractive to travellers, in the form of, for example, an expanded urban rail transit [1]. Tram transport has many advantages. It is a low-emission transport, which is particularly important in the context of increasing air pollution caused by, among other things, combustion vehicles. Another important advantage is the low cost of travel. The use of separated tram tracks offers the possibility of transport that is largely independent of road conditions and therefore attractive, especially during morning and afternoon rush hours.

Over the past few decades, many cities have gradually moved away from ballasted tram tracks to ballastless designs. It has increased the durability of newly built routes and made it possible to build so-called ”green tracks”, i.e., tracks with layers of humus planted with vegetation, thus increasing the biologically active area of the city and making the tracks more friendly to local residents. However, ballastless structures have their disadvantages, one of which is the increased stiffness of the entire structural system compared to ballasted structures, so that more dynamic impacts in the form of vibration and ground-borne noise generated by passing rail vehicles are transmitted to the tramway environment [2,3,4,5]. Managers of tramway networks deal with this problem in various ways—the most common practice is the use of vibration isolation mats.

Vibration isolation elements aimed at railroad structures have been a subject of many studies. Xu et al. [6] investigated a slab track with a resilient mat and developed a calculation model that reflects the dynamic characteristics of the mat. Zhao et al. [7] carried out computational analysis of phononic crystal vibration isolators in a floating slab track. Wang et al. [8] proposed a new sleeper damping track with elastic side-supporting pads and showed that it can have a similar damping capacity as a floating slab track. Zhang et al. [9] analysed antivibration effect of different subway track beds and proved that the rubber cushion floating trackbed had the best vibration reduction performance. Xu et al. [10] carried out theoretical and experimental research on a multi-layer track structure with tuned slab dampers. Cai et al. [11] performed an experimental study on long elastic sleeper tracks used in subways and demonstrated that the vibrations of the track bed were positively mitigated. Cheng et al. [12] proposed an inverter-enhanced dynamic vibration absorber that improves the low-frequency vibration mitigation performance of the floating slab track. Montella et al. [13] investigated an innovative floating slab track system with rubber-based elastic elements. Dudkin et al. [14] examined different ways of reducing noise and vibration in tram track structures, e.g., the use of welded rail strings in combination with temperature compensators.

Resilient vibration isolation elements are usually produced from elastomeric materials such as rubber or polyurethane. However, to reduce the consumption of raw materials while at the same time making use of waste materials that are difficult to dispose of, recycled rubber obtained from deconstructed tires has been successfully applied in the research on elastic vibration isolators [15,16,17,18,19]. A similar approach has also been used by the authors of this paper in their previous studies on resilient elements manufactured from end-of-life tires [20,21,22].

The authors have studied vibration isolation mats, mainly focusing on ballasted track systems, where underballast mats (UBMs) are commonly used. In [21], they analysed the influence of the experimentally determined fatigue strength of UBMs on the effectiveness of vibration damping from rail traffic. In another work [22], they proposed a testing procedure for resistance to severe environmental conditions of UBMs according to the procedure of EN 17282 [23]. The authors analysed the results of weather resistance tests of mats made of rubber granules, polyurethane, and mineral wool, concluding that the best properties are shown by mats made of rubber granules. Mats made of rubber granules and/or fibres were also discussed in [24], where the authors analysed the effect of the mat thickness on the values of their static and dynamic characteristics.

This paper refers to the previously mentioned research works on UBMs investigated according to EN 17282 [23], except that it refers to a different standard—DIN 45673-7 [25] and a different type of mat—under slab mat (USM). The purpose of this study is to explore and provide an overview of the use of recycled under slab vibration isolation mats in ballastless tram track structures. The focus is put on the description of test procedures for the relevant parameters according to the German standard DIN 45673-7 [25], which is the most widely used standard in regard to USMs. Moreover, it contains a discussion on the practical application of the test results of static and dynamic characteristics in the design process by analysing deflections in two reference tram track structures (with concrete slabs or longitudinal beams). An important aspect of this study that the authors would like to highlight is a sustainable approach towards the reuse of a waste material—SBR (styrene-butadiene rubber) granulate and fibres obtained from recycled end-of-life car tires—which is applied here for manufacturing the tested USMs.

Many design requirements or technical projects specify the value of the USM thickness, which may be dictated by important technical issues, such as the limits of the structure gauge or the minimum thickness of the track slab. Another value that is sometimes specified in the requirements is the minimum density of the mat, which is related to the porosity of the material and its possible impact on resistance to weather conditions (water and frost).

It is also worth noting that the determination of static and dynamic elastic characteristics of elastomer materials for railway track systems is conducted by a relatively small number of specialized laboratories around the world. Therefore, the process of testing them by manufacturers is time- and cost-consuming. Most production plants do not have specialized equipment for measuring these parameters. However, such plants do have the ability to measure other parameters correlated with bedding moduli, such as density and thickness, within the factory production control. For such companies, the database of laboratory test results presented in this paper may be used to improve the production process by reducing the cost and time of testing the prototypes.

In this study, the authors focus on the application and engineering use of static bedding moduli values, in terms of predicting maximum deflections of the rail and the floating slab system (with a concrete slab and longitudinal beams), since the use of dynamic bedding moduli has already been analysed by the authors within the framework of vibration mitigation level analyses—transmissibility function (TR) and insertion loss (IL) values—with regard to UBMs in ballasted track structures [24] and with regard to USMs and the embedded block system (EBS) system in ballastless tracks in a railroad tunnel [26].

The authors have also performed service life tests (fatigue strength and resistance to severe environmental conditions) of prototype recycled rubber-based materials, which showed the absence of significant damage and a small (up to 10%) variation in static and/or dynamic parameters after the test relative to pre-test values. These tests are not analysed in detail in this paper due to the focus mainly on the relationship between technical parameters (thickness and density) and static and dynamic properties and their impact on the operation of the tramway track structure.

## 2. Testing of Essential Parameters of USMs According to DIN 45673-7

Construction materials should exhibit properties suitable for the purpose of their use, which in many cases are defined by relevant standards. For the identification of the essential parameters (static and dynamic elastic characteristics) of vibration isolation mats, the reference document most widely used to date (worldwide and in Poland) for this purpose—the German standard DIN 45673-7 [25]—was applied in this study.

From the point of view of the vibration isolation function of USMs, the most essential properties are static and dynamic bedding moduli. They affect the vibration isolation effectiveness of the mat and the values of rail deflections. Mats with higher values of bedding moduli isolate the track system less effectively than softer types. It cannot be neglected, however, that the use of soft mats leads to bigger vertical rail deflections, which, on the other hand, induces high stress in the structural elements of the track system and reduces their fatigue strength.

Static bedding modulus (*C*_stat_) of USM is calculated as a ratio of the static load of a given value applied to the sample of a given cross-sectional area (stress) to the deflection of this sample. It describes the rail deflection under the standing train and affects the vertical deflection of the whole track structure. The dynamic bedding modulus (*C*_dyn_) of USM is calculated as a ratio of the dynamic load of a given value and frequency applied to the sample of a given cross-sectional area (stress) to the deflection of this sample. It describes the rail deflection under the moving train and affects the dynamic deflections of the track system and the vibration isolation effectiveness.

**Figure 1:**
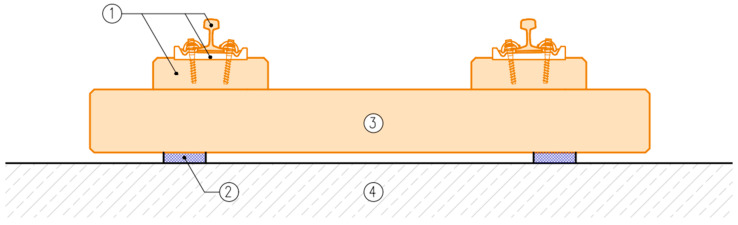
Tram track structure in a mass–spring system: 1—Vignoles rail with rail support and rail fastening system, 2—discrete or strip support with USM, 3—concrete track base plate, 4—tunnel invert slab. Elements of the mass–spring system are marked in orange.

**Figure 2:**
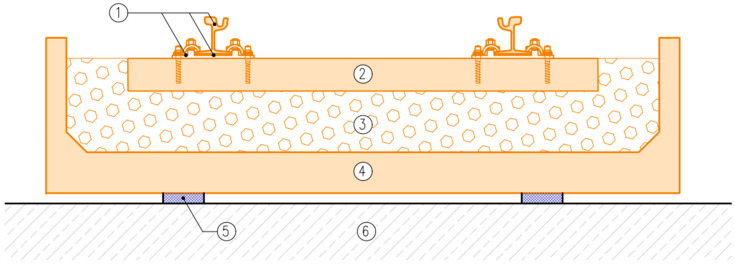
Tram track structure in a mass–spring system: 1—grooved rail with rail fastening system, 2—wooden sleeper, 3—ballast, 4—reinforced concrete trough, 5—discrete or strip support with USM, 6—tunnel invert slab. Elements of the mass–spring system are marked in orange.

**Figure 3:**
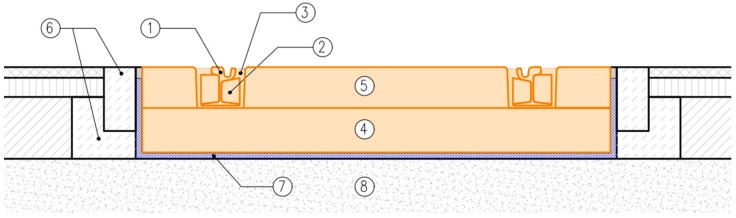
Tram track structure in a mass–spring system: 1—grooved rail, 2—rail web filler block, 3—embedded rail system (ERS) grouting, 4—reinforced concrete track base plate, 5—concrete filling, 6—kerbstone with concrete bench, 7—continuous support with USM, 8—consolidated formation. Elements of the mass–spring system are marked in orange.

DIN 45673-7 [25] describes test methods for elastic materials used in mass–spring systems (floating slab system). It distinguishes three ways of supporting the elements of a mass–spring system:

- Discrete support (Figure 1 and Figure 2);
- Strip support (Figure 1 and Figure 2);
- Continuous support (Figure 3).

There are many possible variants of supports of the elements used in the mass–spring system (floating slab system) and various ways of its design, so it is not possible to determine the impacts transmitted to the vibration isolation mat in a universal way (as is the case of UBMs according to EN 17282 [23])—they should be determined individually for each design solution.

## 3. Laboratory Test Results

### 3.1. USM Samples and Test Stand

In the laboratory of the Faculty of Civil Engineering of the Warsaw University of Technology, selected parameters of prototype vibration isolation mats based on recycled end-of-life car tires were determined. The USM samples were produced from SBR granulate and fibres obtained from mechanical shredding of waste tires, glued with a polyurethane adhesive.

**Figure 4:**
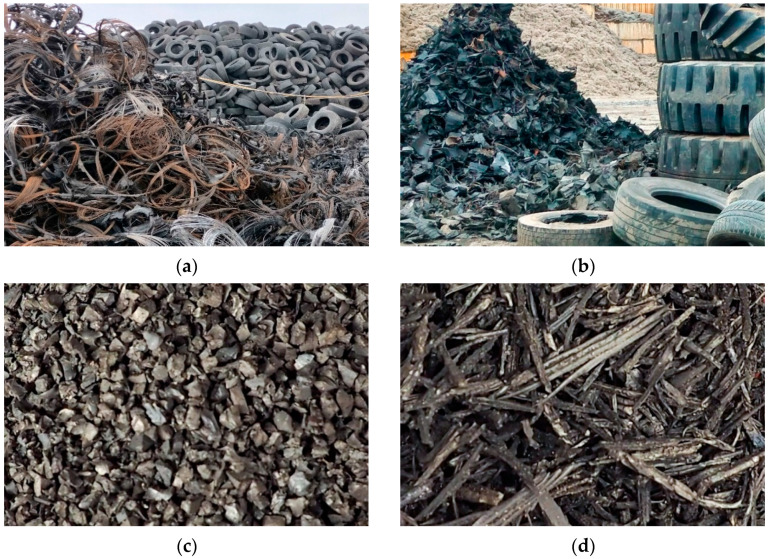
Recycling of end-of-life tires: (**a**) mechanically removed steel (for recycling) against the background of a pile of tires at the storage yard; (**b**) mechanically shredded tires (left side of the photograph); (**c**) SBR granulate; (**d**) SBR fibres.

The recycling process of tires is shown in Figure 4. Mats based on recycled rubber, i.e., composite materials composed of SBR granules (Figure 4c) and fibres (Figure 4d), are bonded together at the stage of the manufacturing process using polyurethane adhesive.

As part of the identification study of static and dynamic elastic characteristics of prototype vibration isolation mats, 39 samples of USMs were tested (3 samples for each material). SBR-based samples had an additional protective layer of navy blue-coloured geotextile on one side. The tested samples had dimensions of 300 mm × 300 mm × 10 mm, with a tolerance of ±5 mm for the width and length and ±0.5 mm for the thickness.

**Figure 5:**
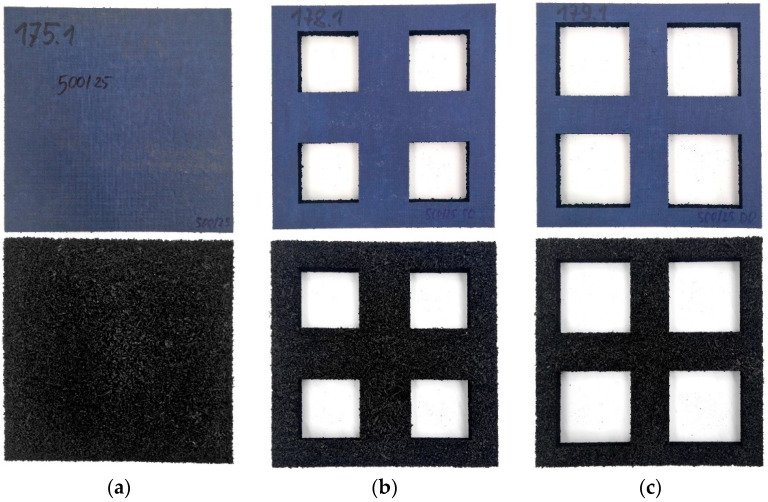
Photographs of selected USM samples with varying hole sizes: (**a**) Sample no. 175.1; (**b**) Sample no. 178.1; (**c**) Sample no. 179.1.

Mats of varying thickness, density, and space-filling (mats with holes), made of rubber fibres and granules, were tested. The parameters of the mats are specified in Table 1, and the differentiation of the hole size for three mats with the same density and thickness is presented in Figure 5.

**Figure 6:**
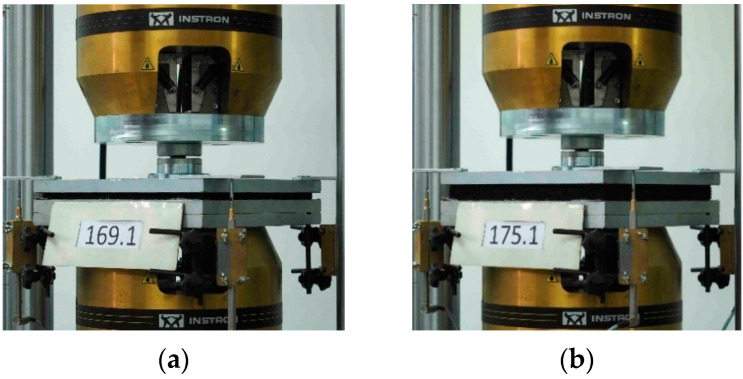
Test stand for the determination of static and dynamic elastic characteristics of USMs: (**a**) Sample no. 169.1 (10 mm); (**b**) Sample no. 175.1 (25 mm).

Examples of test stand configurations according to DIN 45673-7 [25] for testing USMs are shown in Figure 6. For each material (no. 169–181), three samples were tested (marked with no. 1–3 specified after a dot). The test stand consisted of a universal testing machine Instron 8802 (Figure 6; Instron, Norwood, MA, USA) with two steel plates: a bottom support plate 320 mm × 320 mm and a top plate–flat. During the tests, displacements of four points were measured using a test kit consisting of four inductive displacement transducers WA-T (by HBM, Hottinger Baldwin Messtechnik GmbH, Darmstadt, Germany), a signal conditioning system Spider8 and a software Catman AP (version 3.4).

### 3.2. Results for Three Samples of USM No. 175

**Figure 7:**
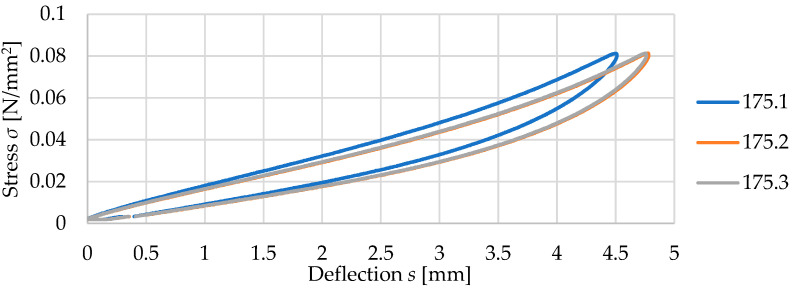
Static elastic characteristics of three samples of USM no. 175.

**Figure 8:**
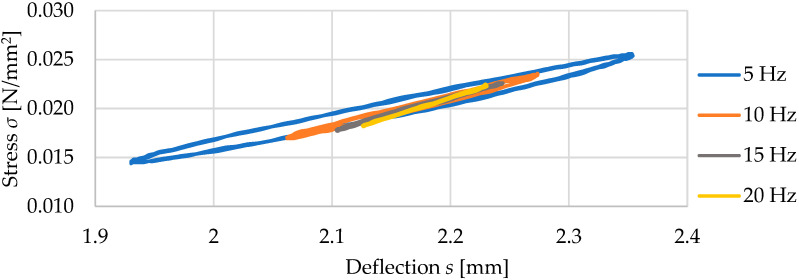
Dynamic elastic characteristics of USM sample no. 175.1 at various load frequencies (initial static load of 0.02 N/mm^2^).

Three vibration isolation mat samples (no. 175.1, 175.2, and 175.3) with dimensions of 300 mm × 300 mm × 10 mm and density of 500 kg/m^3^ were tested. Figure 7 and Figure 8 present static and dynamic elastic characteristics of tested samples in the form of stress–deflection curves. The determined values of the static and dynamic bedding moduli are summarized in Table 2 and Table 3.

It can be noticed that the results obtained for three samples of USM no. 175 are very similar, and the standard deviation values are small both in statics and dynamics. For other tested mats (see Table 1), the situation is analogous, and therefore, further considerations are based on mean values calculated for three samples of each mat.

### 3.3. Influence of USM Thickness on Its Elastic Characteristics

This section focuses on the comparison of results obtained for vibration isolation mats with the same density, the same way of space-filling (no holes), and different thicknesses. The results are summarized in the two following subsections.

#### 3.3.1. USM Samples with a Density of 600 kg/m^3^

**Figure 9:**
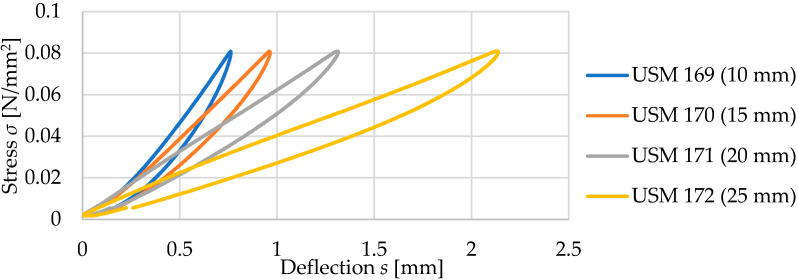
Comparison of the static elastic characteristics of four samples with a density of 600 kg/m^3^.

Tested samples with a density of 600 kg/m^3^ are characterized by a decrease in the value of the static bedding modulus *C*_stat_ with an increase in the thickness of the material, regardless of the range of assessed loads (Table 4). It is also worth noting a significant increase in deflection values with increasing thickness—for a 10 mm thick sample at a stress of 0.05 N/mm^2^, it is about 0.52 mm, and for a 25 mm thick sample at the same stress, it is 1.30 mm (Figure 9).

**Figure 10:**
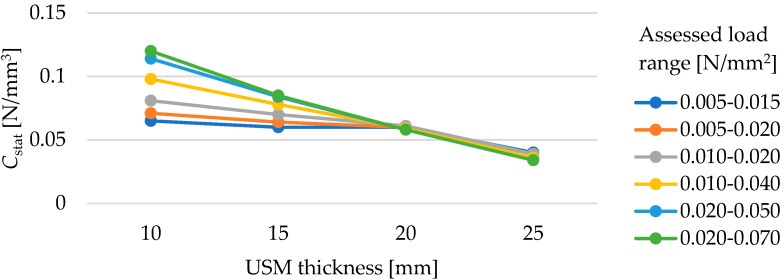
Influence of USM thickness on its static bedding modulus at varying load ranges (for samples with a density of 600 kg/m^3^).

Figure 10 shows how the USM thickness affects its static bedding modulus depending on the assessed load range. The dependence of the value of the static bedding modulus in the range of assessed loads of 0.02–0.07 N/mm^2^ on the mat thickness is approximately linear. The lower and narrower the range of assessed loads, the smaller the effect of the increase in thickness on the decrease in the value of the static bedding modulus, which is evident given the almost linear stress–deflection plot (Figure 9) for the thickness of 25 mm and the increase in stiffness with decreasing mat thickness up to 10 mm (visible nonlinearity).

**Figure 11:**
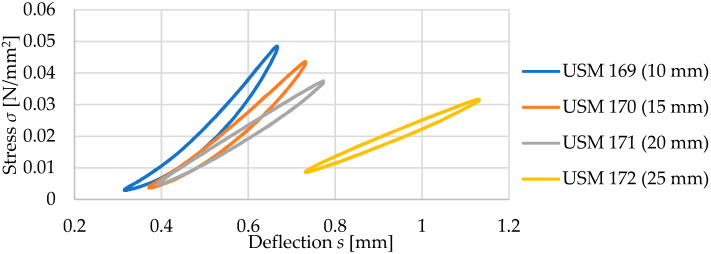
Comparison of the dynamic elastic characteristics at 5 Hz of four samples with a density of 600 kg/m^3^ (initial static load of 0.02 N/mm^2^).

Dynamic elastic characteristics of the tested mats are presented in Figure 11 and in Table 5. As the thickness of the test samples increases, a decrease in the value of the dynamic bedding modulus *C*_dyn_ is observed, regardless of the frequency at which the test was conducted. It is worth noting the values of deflections of individual mats under dynamic loading. Samples with thicknesses of 10, 15, and 20 mm at a frequency of applied load of 5 Hz exhibit deflections within the range of 0.30–0.75 mm, while the deflections of the 25 mm thick samples are within the range of 0.70–1.15 mm (higher initial deflection from the initial static load of 0.02 N/mm^2^).

**Figure 12:**
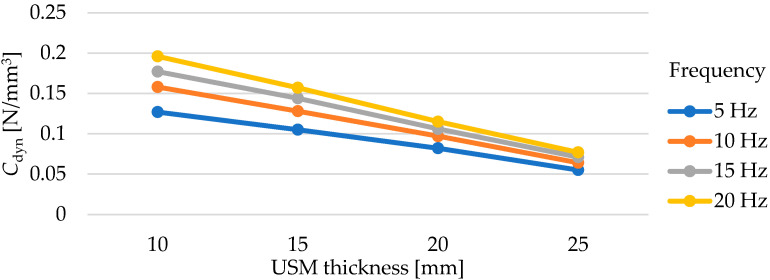
Influence of USM thickness on its dynamic bedding modulus tested at various frequencies (for samples with a density of 600 kg/m^3^).

**Figure 13:**
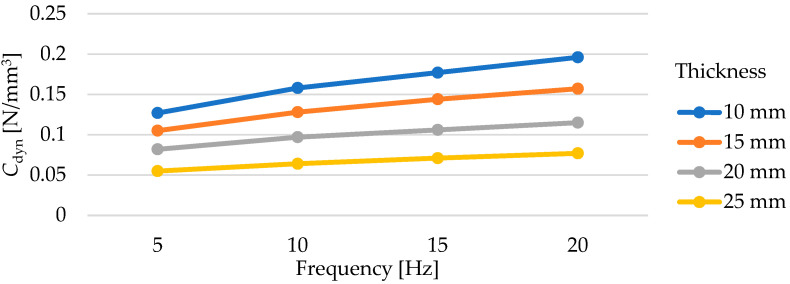
Influence of load frequency on the dynamic bedding modulus of USMs with varying thicknesses and a density of 600 kg/m^3^ (initial static load of 0.02 N/mm^2^).

The dependence of the value of the dynamic bedding modulus on the mat thickness is approximately linear (Figure 12). As the test frequency increases, both the value of the dynamic bedding modulus and the value of the dynamic stiffening ratio increase (Figure 13). This relationship is approximately linear in the range from 5 to 20 Hz.

#### 3.3.2. USM Samples with a Density of 500 kg/m^3^

**Figure 14:**
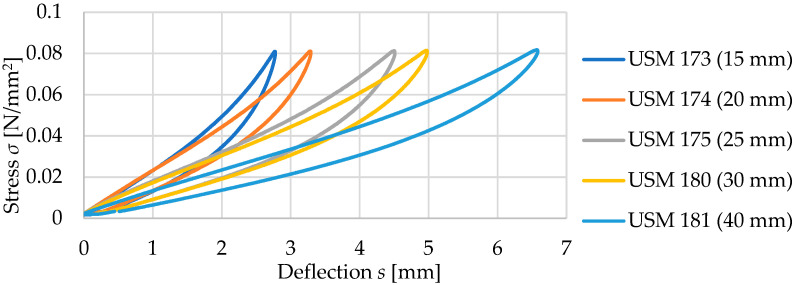
Comparison of the static elastic characteristics of five samples with a density of 500 kg/m^3^.

Tested samples with a density of 500 kg/m^3^ are characterized by a decrease in the value of the static bedding modulus *C*_stat_ with an increase in the thickness of the material, regardless of the range of assessed loads (Table 6). It is also worth noting a significant increase in deflection values with increasing thickness—for a 15 mm thick sample at a stress of 0.05 N/mm^2^, it is about 2.05 mm, and for a 40 mm thick sample at the same stress, it is 4.56 mm (Figure 14).

**Figure 15:**
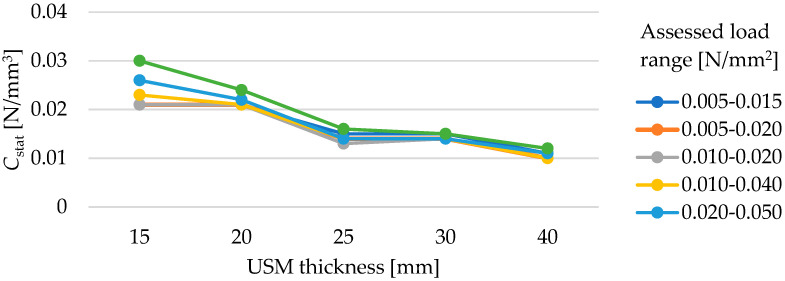
Influence of USM thickness on its static bedding modulus at varying load ranges (for samples with a density of 500 kg/m^3^).

Figure 15 shows how the USM thickness affects its static bedding modulus depending on the assessed load range. The dependence of the value of the static bedding modulus on the mat thickness is not linear for any of the assessed load ranges. The results obtained for the 25 mm thick sample are similar within all load ranges to the results for the 30 mm thick sample (the difference of 0.001 N/m^3^ for 5 out of 6 assessed load ranges).

**Figure 16:**
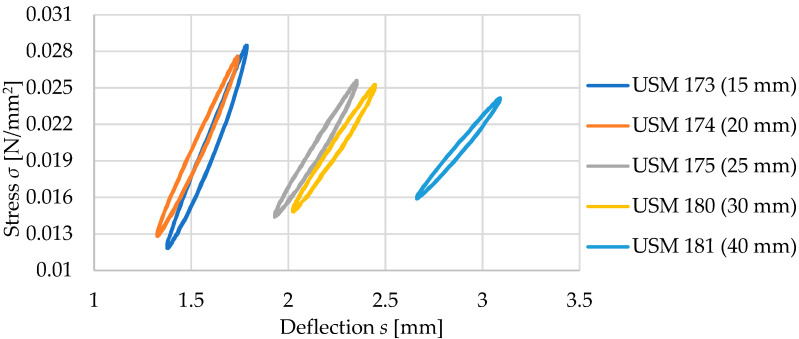
Comparison of the dynamic elastic characteristics at 5 Hz of five samples with a density of 500 kg/m^3^ (initial static load of 0.02 N/mm^2^).

Dynamic elastic characteristics of the tested mats are presented in Figure 16 and in Table 7. As the thickness of the test samples increases, a decrease in the value of the dynamic bedding modulus *C*_dyn_ is observed, regardless of the frequency at which the test was conducted. It is worth noting the values of deflections of individual mats under dynamic loading. Samples with thicknesses of 15 and 20 mm at a frequency of applied load of 5 Hz exhibit deflections within the range of 1.30–1.80 mm, 25- and 30-mm thick samples—within the range of 1.90–2.50 mm, while the deflections of the 40 mm thick sample are within the range of 2.60–3.10 mm (higher initial deflection from the initial static load of 0.02 N/mm^2^).

**Figure 17:**
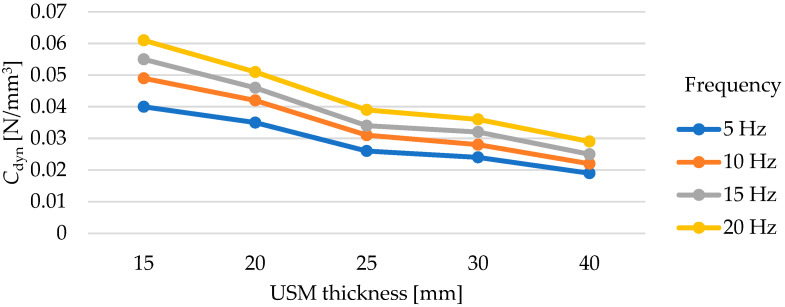
Influence of USM thickness on its dynamic bedding modulus tested at various frequencies (for samples with a density of 500 kg/m^3^).

The dependence of the value of the dynamic bedding modulus on the mat thickness cannot be described as linear (Figure 17). The test results for a 25 mm thick sample are very similar to those for a 30 mm thick mat—the differences are at a level of 0.002–0.003 N/mm^3^.

**Figure 18:**
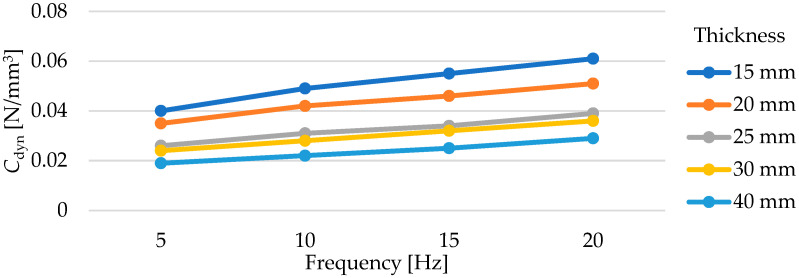
Influence of load frequency on the dynamic bedding modulus of USMs with varying thicknesses and a density of 500 kg/m^3^ (initial static load of 0.02 N/mm^2^).

As the test frequency increases, both the value of the dynamic bedding modulus and the value of the dynamic stiffening ratio increase (Figure 18). This relationship is approximately linear in the range from 5 to 20 Hz.

### 3.4. Influence of USM Density on Its Elastic Characteristics

**Figure 19:**
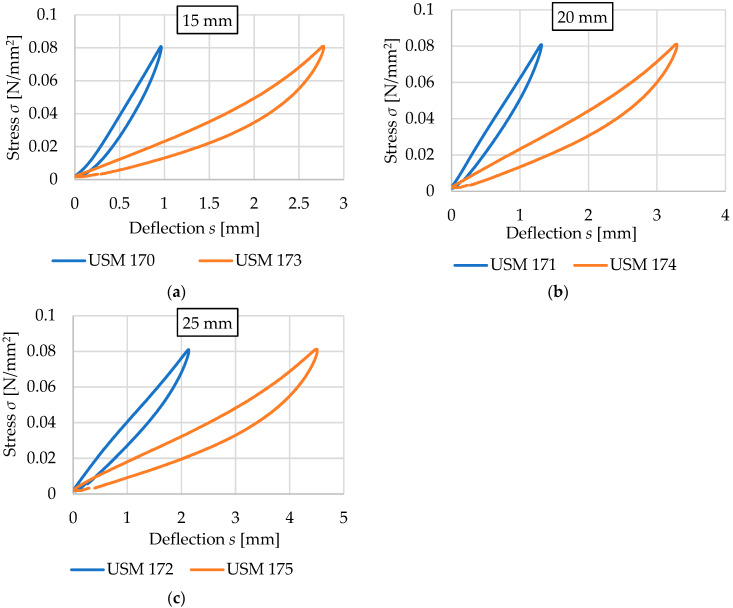
Comparison of the static elastic characteristics for USM samples with corresponding thicknesses and varying densities (blue curves—density 600 kg/m^3^, orange curves—density 500 kg/m^3^): (**a**) 15 mm thick samples; (**b**) 20 mm thick samples; (**c**) 25 mm thick samples.

**Figure 20:**
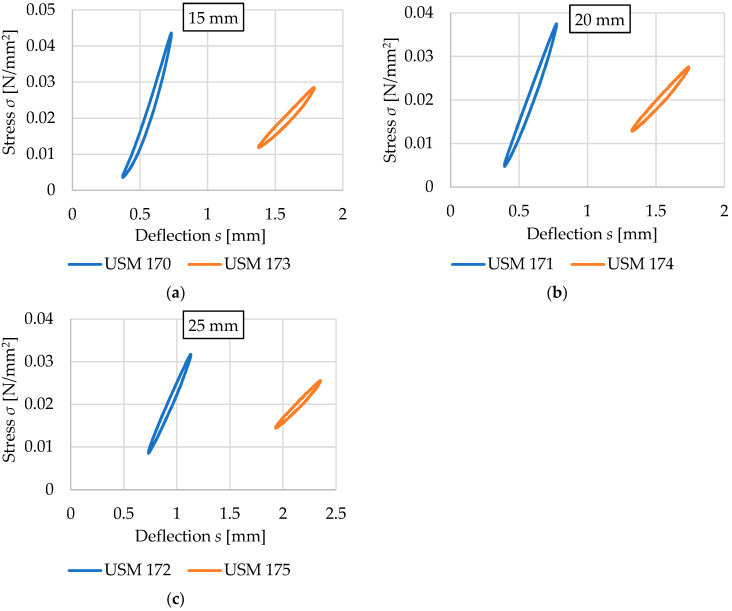
Comparison of the dynamic elastic characteristics at 5 Hz for USM samples with corresponding thicknesses and varying densities (blue curves—density 600 kg/m^3^, orange curves—density 500 kg/m^3^): (**a**) 15 mm thick samples; (**b**) 20 mm thick samples; (**c**) 25 mm thick samples.

This section focuses on the comparison of results obtained for vibration isolation mats with the same thickness, the same way of space-filling (no holes), and different densities. The results are summarized in Figure 19 and Figure 20 and Table 8 and Table 9.

USM samples with a density of 600 kg/m^3^ are characterized by significantly higher values of both static (Table 8) and dynamic (Table 9) bedding moduli compared to samples with a density of 500 kg/m^3^, and thus have lower vibration isolation properties but also lower deflection values. The percentage differences of the compared values decrease with increasing thickness of the samples.

The largest differences are observed in the static bedding modulus for 15 mm thick mats (the values for USM with a density of 600 kg/m^3^ are 3.00–3.62 times higher than those for 500 kg/m^3^). The smallest differences are observed in the results of the dynamic bedding modulus test for 25 mm thick mats (the values for USM with a density of 600 kg/m^3^ are 2.05–2.23 times greater than the values for 500 kg/m^3^).

### 3.5. Influence of Space Filling in USM on Its Elastic Characteristics

**Figure 21:**
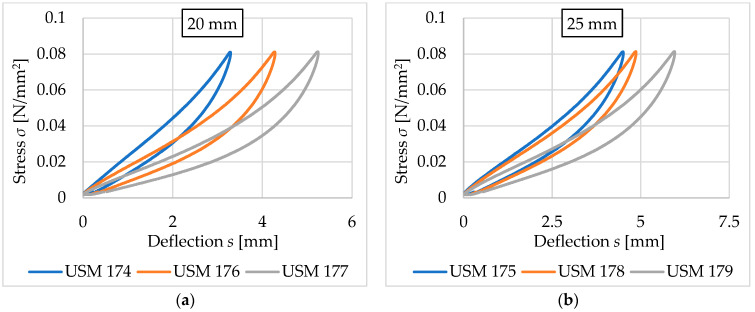
Comparison of the static elastic characteristics for USM samples with corresponding thicknesses and varying space filling (blue curves—no holes, orange curves—medium holes, grey curves—large holes): (**a**) 20 mm thick samples; (**b**) 25 mm thick samples.

**Figure 22:**
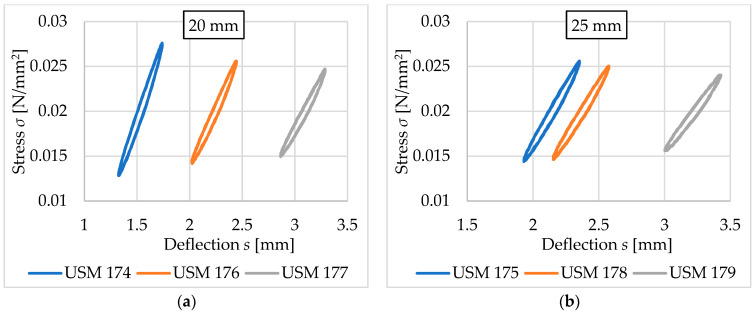
Comparison of the dynamic elastic characteristics at 5 Hz for USM samples with corresponding thicknesses and varying space filling (blue curves—no holes, orange curves—medium holes, grey curves—large holes): (**a**) 20 mm thick samples; (**b**) 25 mm thick samples.

This section focuses on the comparison of results obtained for vibration isolation mats with the same thickness, the same density (500 kg/m^3^), and different ways of space filling (no holes, medium holes, and large holes). The results are summarized in Figure 21 and Figure 22 and Table 10 and Table 11.

Holes in the tested samples reduce the values of both static (Table 10) and dynamic (Table 11) bedding moduli and increase deflection. The static bedding modulus can be reduced up to 33–52% (depending on the assessed load range) in the 20 mm thick mat and up to 19–40% in the 25 mm thick mat. The reduction of the dynamic bedding modulus is at the level of 31–37% (depending on the load frequency) in the thinner mat and 18–23% in the thicker one. The 20 mm thick mat with large holes has better vibration isolation properties than the 25 mm thick mat without holes.

### 3.6. Conclusions from Laboratory Tests

Test results of static and dynamic elastic characteristics of selected USM samples were analysed by comparing them with each other in three different ways:

- Comparison of test results of USMs with equal density and varied thickness;
- Comparison of test results of USMs with equal thickness and varied density;
- Comparison of test results of USMs with equal thickness and density but with varied space filling (no holes, medium holes, and large holes).

The analysis of the results presented in Section 3 leads to the following conclusions:

- As the thickness of the USM samples increases (while maintaining the same density and the same way of space filling), the values of the static and dynamic bedding moduli decrease, and this relationship is approximately linear.
- As the density of the USM samples increases (while maintaining the same thickness and the same way of space filling), the values of the static and dynamic bedding moduli increase;
- As the load frequency in the test of dynamic characteristics of the USM sample increases, the value of the dynamic bedding modulus and the value of the dynamic stiffening ratio increase;
- Making holes in the USM samples causes a decrease in the value of static and dynamic bedding modulus.

The dependence of the stress on the deflection induced by this stress for the tested samples of vibration isolation mats made of rubber granules and fibres is approximately linear almost in the entire tested range, which translates into a more predictable behaviour of such mats in tram track systems, relative to polyurethane-based mats [27] (regardless of the railroad subsystem or the loads resulting from the possible variation of the tram track structure or the variation of the tram rolling stock).

## 4. Application of Experimental Test Results in the Analysis of Tram Track Systems with USMs

### 4.1. Assumptions

In order to correctly determine the range of assessed static and dynamic characteristics of USMs applied in tram track structures, it is necessary to determine the values of stresses transferred to the vibration isolation mat arising from the tramway traffic loads. In this study, maximum stresses under the floating slab system were determined according to the procedure of the European standard EN 16432-2 [28].

**Figure 23:**
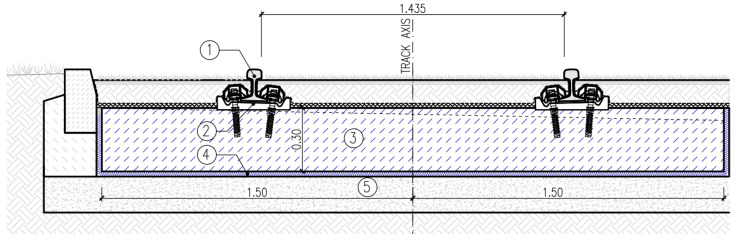
Type 1 track structure: 1—Vignoles rail 49E1, 2—rail support and rail fastening system, 3—concrete slab, 4—USM, 5—protective layer of unbound aggregate mixture.

**Figure 24:**
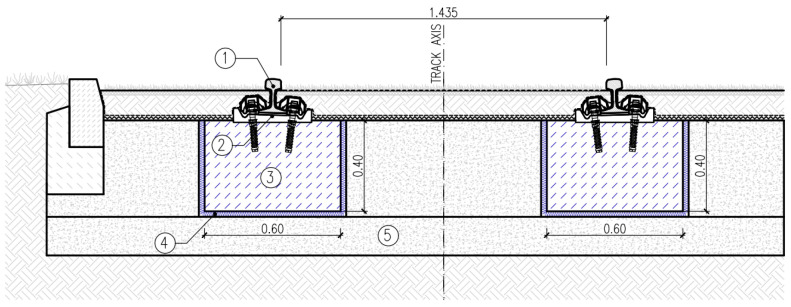
Type 2 track structure: 1—Vignoles rail 49E1, 2—rail support and rail fastening system, 3—concrete beam, 4—USM, 5—protective layer of unbound aggregate mixture.

Two types of tram track structures in the mass–spring system were analysed, with the same rail fastening system and different ways of transferring loads to the USM:

- Type 1—structure with discrete supports and discrete fastening of the rail to the floating slab system in the form of a concrete slab (Figure 23);
- Type 2—structure with discrete supports and discrete fastening of the rail to the floating slab system in the form of longitudinal concrete beams (Figure 24).

In both types of track structures, Vignoles rails 49E1 made of steel R260 with W-Tram rail fastening system were used, with supports spacing of 0.75 m, according to [29]. Both floating slab system types were made of cement concrete C30/37, and protective layers of unbound aggregate mixture with a grain size of 0/31.5 mm were used as substructures.

**Figure 25:**
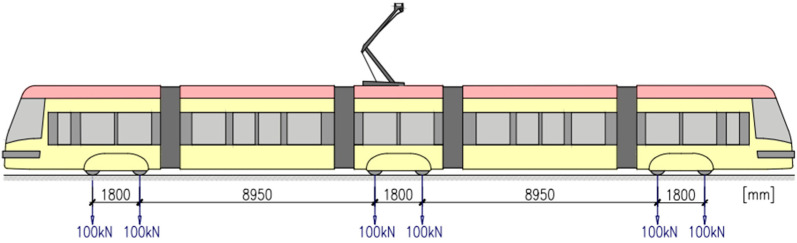
Reference tram vehicle—PESA “Swing” 120Na.

As a reference tram vehicle, wheelbases and bogie distances of the most common type of tram used in Warsaw—PESA “Swing” 120Na (Figure 25)—were adopted. The value of a single axle load of a wheelset was adopted as the maximum allowed by the Polish regulations [30], i.e., 100 kN.

Calculations of rail deflections, reactions to the track structure, and stresses under the track structure were carried out in accordance with the methodology presented in EN 16432-2 [28], which is based on Winkler-Zimmerman theory. It is assumed that a rail with discrete supports can be regarded as a beam on an elastic substrate, the equivalent length of which is determined according to equation:

(1) $$L_{el}=\sqrt[{4}]{\frac{4\cdot E_{R}\cdot I_{R}\cdot a}{C_{tot}}},$$

where $E_{R}$ and $I_{R}$ are the elastic modulus of the material and the moment of inertia of the rail, respectively, a is the spacing of rail supports, $C_{tot}$ is the static stiffness of the rail support.

Then, knowing the load on the rail $Q_{0}$, the deflection at the point x=0 can be determined:

(2) $$y_{0}=\frac{Q_{0}\cdot a}{2\cdot C_{tot}\cdot L_{el}}.$$

The effect of additional forces (n) located at other distances $x_{i}$ from the section x=0, is calculated from the relationship:

(3) $$n_{i}=\frac{\sin\left(\xi_{i}\right)+\cos\left(\xi_{i}\right)}{e^{\xi_{i}}},\;\;\;\;\;\;\;\;\;\xi_{i}=\frac{x_{i}}{L_{el}},$$

(4) $$y=y_{0}+\sum_{i=1}^{n}\frac{Q_{0}\cdot a}{2\cdot C_{tot}\cdot L_{el}}\cdot \eta_{i}.$$

Knowing the deflections of the rail, the forces acting on individual rail supports can be determined:

(5) $$F_{i}=-y_{i}\cdot C_{tot}\;.$$

Reactions from the rail supports are transferred to the tram floating slab system (Type 1 or 2 structure), which is laid on USM characterized by the static bedding modulus $C_{stat}$ or the dynamic bedding modulus $C_{dyn}$. In this case, again the Winkler–Zimmerman theory is applied, and the equivalent length of the beam is determined, taking into account the geometric characteristics of Type 1 and Type 2 structures under one rail, according to the formula:

(6) $$L_{el,P}=\sqrt[{4}]{\frac{4\cdot E_{P}\cdot I_{P}}{b_{p}\cdot C_{stat}}},$$

where $E_{P}$ is the elastic modulus of the structural material, $I_{P}$ is the moment of inertia of the structure section, $b_{p}$ is the width cooperating with one rail, $C_{stat}$ is the static bedding modulus of the reference USM.

The static stiffness of a section of USM corresponding to one rail support can be calculated from the relationship:

(7) $$C_{M}=C_{stat}\cdot b_{P}\cdot a\;.$$

In order to determine the deflections of the floating slab system, a relationship analogous to that for determining the deflections of the rail was used, taking into account a larger range of the influence of the supports, namely from i=−10 to i=25, according to the relationship:

(8) $$y_{P}\left(x\right)=\sum_{i=-10}^{25}\left[\left(\frac{F_{i}\cdot a}{2\cdot C_{M}\cdot L_{el,P}}\right)\cdot \eta \left(\frac{x-i\cdot a}{L_{el,P}}\right)\right],$$

where:

(9) $$\begin{array}{c} \eta \left(\xi \right)=-\frac{\sin\left(\xi \right)+\cos\left(\xi \right)}{e^{\xi }}\;\;\;\;\;\;\;\;\;\;\;\;\;\;for\;\;\;\xi \geq 0,\\ \eta \left(\xi \right)=-\frac{\sin\left(-\xi \right)+\cos\left(-\xi \right)}{e^{-\xi }}\;\;\;\;\;\;\;for\;\xi <0,\\ \xi \left(x\right)=\frac{x}{L_{el,P}}\;. \end{array}$$

In order to determine stresses under the floating slab system in two types of track structures with USMs, stresses from the dead weight (g) of the structure and stresses from the service load (q) must be taken into account:

(10) $$\sigma_{max}=\sigma_{g}+\sigma_{q}\;,$$

(11) $$\sigma_{q}=y_{P}\cdot C_{stat}\;.$$

### 4.2. Analysis of Tram Track Structures with USMs

The potential application of three selected vibration isolation mats made of rubber granules and fibres with elastic characteristics described in Section 3 was analysed in relation to two types of tram track structures presented in Section 4.1.

USMs selected for the analysis:

- USM no. 173—density 500 kg/m^3^, thickness 15 mm;
- USM no. 175—density 500 kg/m^3^, thickness 25 mm;
- USM no. 181—density 500 kg/m^3^, thickness 40 mm.

Based on the test results obtained for USMs no. 173, 175, and 181 (see Section 3), as well as the stress ranges acting on the vibration isolation mat specified in Section 3, the static bedding moduli of USMs were determined for both types of tram track structures. Aan example of such calculations is presented below for one selected sample—USM 175.1, and the results for all considered USM samples are summarized in Table 12, Table 13 and Table 14.

**Figure 26:**
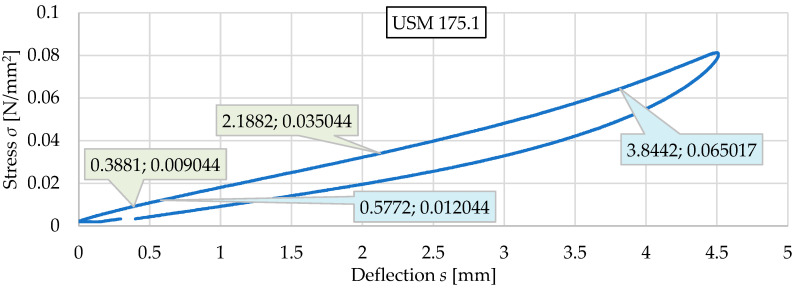
Static elastic characteristics of USM sample no. 175.1.

Figure 26 shows a diagram of static elastic characteristics of USM sample no. 175.1.

Based on this diagram, the values of static bedding moduli are calculated as follows:

(12) $$\begin{array}{c} C_{stat,Type1}=\frac{0.035044-0.009044}{2.1882-0.3881}=0.0144\;N/mm^{3}\\ C_{stat,Type2}=\frac{0.065017-0.012044}{3.8442-0.5772}=0.0162\;N/mm^{3} \end{array}$$

Similar calculations were performed for other USM samples and the summarized results are presented in Table 12, Table 13 and Table 14.

**Figure 27:**
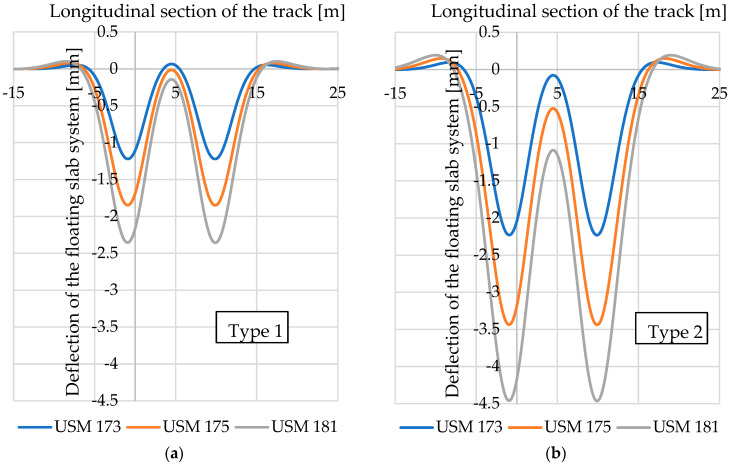
Diagram of the floating slab system deflections in two types of tram track structures with USMs: (**a**) Type 1 (concrete slab); (**b**) Type 2 (concrete beams).

**Figure 28:**
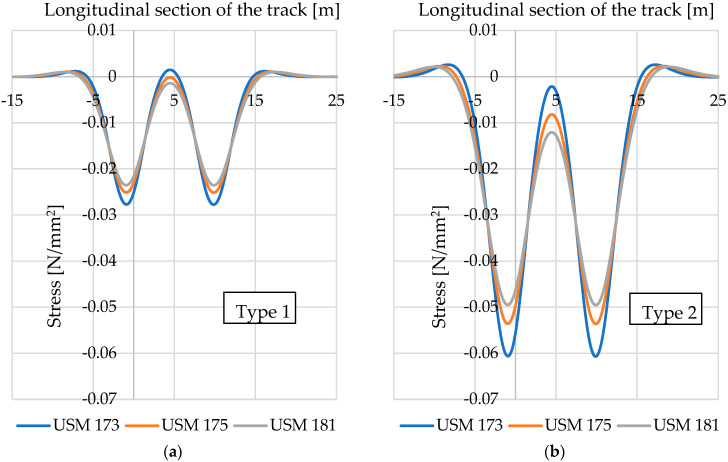
Diagram of compressive stresses under the floating slab system in two types of tram track structures with USMs: (**a**) Type 1 (concrete slab); (**b**) Type 2 (concrete beams).

Using the formulas described in Section 4.1 and the values of static bedding moduli specified in Table 12, Table 13 and Table 14, it is possible to determine deflections of the floating slab system after the application of each considered vibration isolation mat. Figure 27 shows diagrams of the floating slab system deflections, and Figure 28—compressive stresses under the floating slab system, determined for two types of tram track structures with USMs of varying thickness.

It can be noticed that the application of USMs of higher thickness (and thus a lower value of the static bedding modulus) in both reference tram track structures results in an increase in the deflections of the floating slab system and a simultaneous decrease in the maximum compressive stress under the floating slab system.

It is also worth noting the differences in the values of floating slab system deflections and compressive stress between the two analysed types of tram track structure. For the same USM type, in the structure of Type 2, both deflections and stresses are almost twice as high as in Type 1.

## 5. Conclusions

In this study, testing procedures for the relevant parameters of under slab vibration isolation mats used in ballastless tram track structures were examined in accordance with the German standard DIN 45673-7, which has been most widely used around the world. The most relevant parameters of USMs include static and dynamic bedding moduli (alternatively: static and dynamic stiffness).

Test results of static and dynamic elastic characteristics according to the procedures of DIN 45673-7 for more than a dozen recycling-based vibration isolation mats made of rubber granules and fibres of varying thickness, density, and space-filling (mats with holes) were presented. Analysis of the test results allows us to draw the following conclusions:

- Increase of the USM thickness (in mats with the same density and the same way of space filling) results in decreasing values of the static and dynamic bedding moduli—the static bedding modulus decreases by 38–72% (depending on the assessed load range) when the mat thickness changes from 10 mm to 25 mm, and the dynamic bedding modulus decreases by 57–60% (depending on the load frequency);
- An increase in the USM density (in mats with the same thickness and the same way of space filling) results in increasing values of the static and dynamic bedding moduli—observed increases of the static bedding moduli are at the level of 112–239% (depending on the mat thickness and the assessed load range), and in the case of the dynamic bedding moduli 103–163% (depending on the mat thickness and the load frequency);
- Increase of the load frequency in the test of USM dynamic characteristics leads to increasing values of the dynamic bedding modulus and the dynamic stiffening ratio;
- Holes in the USM samples lead to decreasing values of the static and dynamic bedding moduli—the static bedding modulus can be reduced up to 33–52% (depending on the assessed load range) in the 20 mm thick mat and up to 19–40% in the 25 mm thick mat, while the dynamic bedding modulus can be reduced up to 31–37% (depending on the load frequency) in the thinner mat and 18–23% in the thicker one;
- The dependence of the stress on the deflection induced by this stress for the tested SBR-based USM samples is approximately linear within almost the entire tested range, which translates into a predictable operation of such an element in the tram track structure.

In the last part of this paper, two types of ballastless tram track structures in the mass-spring system were analysed, and the deflections of the floating slab system and the compressive stresses under the floating slab system were determined. The use of three different SBR-based USMs in these two types of structures was then analysed.

Both of these analyses allow us to conclude that the values of the floating slab system deflections and compressive stresses transferred to the USM can vary significantly depending on the type of structure and the way loads are transferred to the lower layers of the floating slab system. To ensure the correct operation of the entire system during the entire life cycle of the structure, it is necessary to identify at the design stage the range of loads transferred to the vibration isolation mat in order to make an informed selection. Static and dynamic characteristic diagrams or bedding moduli values determined for many ranges of applied and assessed loads and many load frequencies are necessary to properly identify the USM parameters.

For elastomer production plants that have the ability to measure parameters correlated with bedding moduli, the use of the database of laboratory test results presented in this study can give the opportunity to make the production process more efficient and adjust their technical parameters (density and thickness) at the production stage to the requirements of the railway infrastructure manager or the specifics of a particular investment project.

In the authors’ opinion, elastomeric materials derived from recycled car tires, based on SBR granules and fibres, show satisfactory characteristics and are a good solution for sustainable and ecological tram track systems. The proposed reuse of rubber from waste tires as a material of vibration isolation elements in various track systems may be one of the most effective ways for recycling this waste material.

Apart from the analyses presented in this study, the authors have also performed service life tests (fatigue strength and resistance to severe environmental conditions) of prototype recycled rubber-based materials, which showed the absence of significant damage and a small (up to 10%) variation in static and/or dynamic parameters after the test relative to pre-test values. These tests are not analysed in detail in this paper due to the focus mainly on the relationship between technical parameters (thickness and density) and static and dynamic properties and their impact on the operation of the tramway track structure. 

Future studies will focus on evaluating the effect of prototype mats of varying thicknesses and densities (which implies varying values of dynamic moduli detected in this article) on the vibration isolation of various solutions of tramway track structures (e.g., beams, slab—analysed in this article) in real trackside measurements of the level of vibrations transmitted to the environment of the tramway route. Moreover, the authors plan to compare the results of tests on the effect of severe weather conditions (water, frost, high temperature) and cyclic dynamic loads (fatigue strength) determined for prototype mats in laboratory tests according to the procedures of DIN 45673-7 on the factual decrease in the effectiveness of vibration isolation of tramway tracks (e.g., dynamic stiffening of the mats) over several years of its service life—i.e., after transferring a certain cumulative axial load (from actual trams) and several winter periods (when alternating freeze–thaw cycles occur).

## Figures and Tables

**Table 1:** Parameters of tested SBR-based USMs.

Material No.	Density [kg/m^3^]	Thickness [mm]	Holes
169	600	10	-
170	600	15	-
171	600	20	-
172	600	25	-
173	500	15	-
174	500	20	-
175	500	25	-
176	500	20	Medium holes
177	500	20	Large holes
178	500	25	Medium holes
179	500	25	Large holes
180	500	30	-
181	500	40	-

**Table 2:** Static bedding modulus of USM no. 175.

Assessed Load Range *σ* [N/mm^2^]	Static Bedding Modulus *C*_stat_ [N/mm^3^]				
	Sample No. 175.1	Sample No. 175.2	Sample No. 175.3	Mean	Standard Deviation
0.005–0.015	0.016	0.014	0.014	0.015	0.001
0.005–0.020	0.015	0.013	0.013	0.014	0.001
0.010–0.020	0.014	0.013	0.013	0.013	0.001
0.010–0.040	0.015	0.013	0.013	0.014	0.001
0.020–0.050	0.015	0.014	0.014	0.014	0.001
0.020–0.070	0.017	0.016	0.016	0.016	0.001

**Table 3:** Dynamic bedding modulus of USM no. 175 (initial static load of 0.02 N/mm^2^).

Frequency *f* [Hz]	Dynamic Bedding Modulus *C*_dyn_ [N/mm^3^]				
	Sample No. 175.1	Sample No. 175.2	Sample No. 175.3	Mean	Standard Deviation
5	0.026	0.025	0.025	0.025	0.001
10	0.031	0.029	0.029	0.030	0.001
15	0.034	0.033	0.033	0.033	0.001
20	0.039	0.037	0.037	0.038	0.001

**Table 4:** Comparison of the static bedding modulus of four samples with a density of 600 kg/m^3^.

Assessed Load Range σ [N/mm^2^]	Static Bedding Modulus Cstat [N/mm^3^]			
	169 (10 mm)	170 (15 mm)	171 (20 mm)	172 (25 mm)
0.005–0.015	0.065	0.060	0.060	0.040
0.005–0.020	0.071	0.064	0.060	0.039
0.010–0.020	0.081	0.070	0.061	0.039
0.010–0.040	0.098	0.078	0.059	0.036
0.020–0.050	0.114	0.084	0.058	0.034
0.020–0.070	0.120	0.085	0.058	0.034

**Table 5:** Comparison of the dynamic bedding modulus of four samples with a density of 600 kg/m^3^.

Frequency *f* [Hz]	Dynamic Bedding Modulus *C*_dyn_ [N/mm^3^]			
	169 (10 mm)	170 (15 mm)	171 (20 mm)	172 (25 mm)
5	0.127	0.105	0.082	0.055
10	0.158	0.128	0.097	0.064
15	0.177	0.144	0.106	0.071
20	0.196	0.157	0.115	0.077

**Table 6:** Comparison of the static bedding modulus of five samples with a density of 500 kg/m^3^.

Assessed Load Range *σ* [N/mm^2^]	Static Bedding Modulus *C*_stat_ [N/mm^3^]				
	173 (15 mm)	174 (20 mm)	175 (25 mm)	180 (30 mm)	181 (40 mm)
0.005–0.015	0.021	0.021	0.015	0.015	0.011
0.005–0.020	0.021	0.021	0.014	0.014	0.010
0.010–0.020	0.021	0.021	0.013	0.014	0.010
0.010–0.040	0.023	0.021	0.014	0.014	0.010
0.020–0.050	0.026	0.022	0.014	0.014	0.011
0.020–0.070	0.030	0.024	0.016	0.015	0.012

**Table 7:** Comparison of the dynamic bedding modulus of five samples with a density of 500 kg/m^3^.

**Frequency** ***f*** **[Hz]**	**Dynamic Bedding Modulus** ***C*** **_dyn_** **[N/mm^3^]**				
	**173 (15 mm)**	**174 (20 mm)**	**175 (25 mm)**	**180 (30 mm)**	**181 (40 mm)**
5	0.040	0.035	0.026	0.024	0.019
10	0.049	0.041	0.030	0.029	0.022
15	0.055	0.045	0.033	0.032	0.025
20	0.061	0.051	0.038	0.037	0.028

**Table 8:** Comparison of the static bedding modulus of USM samples with corresponding thicknesses and varying densities.

Assessed Load Range *σ* [N/mm^2^]	Static Bedding Modulus *C*_stat_ [N/mm^3^]					
	15 mm		20 mm		25 mm	
	170 (600 kg/m^3^)	173 (500 kg/m^3^)	171 (600 kg/m^3^)	174 (500 kg/m^3^)	172 (600 kg/m^3^)	175 (500 kg/m^3^)
0.005–0.015	0.060	0.021	0.060	0.021	0.040	0.015
0.005–0.020	0.064	0.021	0.060	0.021	0.039	0.014
0.010–0.020	0.070	0.021	0.061	0.021	0.039	0.013
0.010–0.040	0.078	0.023	0.059	0.021	0.036	0.014
0.020–0.050	0.084	0.026	0.058	0.022	0.034	0.014
0.020–0.070	0.085	0.030	0.058	0.024	0.034	0.016

**Table 9:** Comparison of the dynamic bedding modulus of USM samples with corresponding thicknesses and varying densities (initial static load of 0.02 N/mm^2^).

Frequency *f* [Hz]	Dynamic Bedding Modulus *C*_dyn_ [N/mm^3^]					
	15 mm		20 mm		25 mm	
	170 (600 kg/m^3^)	173 (500 kg/m^3^)	171 (600 kg/m^3^)	174 (500 kg/m^3^)	172 (600 kg/m^3^)	175 (500 kg/m^3^)
5	0.105	0.040	0.082	0.035	0.055	0.025
10	0.128	0.049	0.097	0.041	0.064	0.030
15	0.144	0.055	0.106	0.045	0.071	0.033
20	0.157	0.061	0.115	0.051	0.077	0.038

**Table 10:** Comparison of the static bedding modulus of USM samples with corresponding thicknesses and varying space filling.

Assessed Load Range *σ* [N/mm^2^]	Static Bedding Modulus *C*_stat_ [N/mm^3^]					
	20 mm			25 mm		
	174 (No Holes)	176 (Medium Holes)	177 (Large Holes)	175 (No Holes)	178 (Medium Holes)	179 (Large Holes)
0.005–0.015	0.021	0.015	0.010	0.015	0.014	0.009
0.005–0.020	0.021	0.014	0.010	0.014	0.014	0.009
0.010–0.020	0.021	0.014	0.010	0.013	0.013	0.009
0.010–0.040	0.021	0.014	0.011	0.014	0.013	0.010
0.020–0.050	0.022	0.016	0.013	0.014	0.014	0.011
0.020–0.070	0.024	0.018	0.016	0.016	0.016	0.013

**Table 11:** Comparison of the dynamic bedding modulus of USM samples with corresponding thicknesses and varying space filling (initial static load of 0.02 N/mm^2^).

Frequency *f* [Hz]	Dynamic Bedding Modulus *C*_dyn_ [N/mm^3^]					
	20 mm			25 mm		
	174 (No Holes)	176 (Medium Holes)	177 (Large Holes)	175 (No Holes)	178 (Medium Holes)	179 (Large Holes)
5	0.035	0.026	0.022	0.026	0.024	0.020
10	0.041	0.032	0.027	0.030	0.029	0.024
15	0.045	0.036	0.031	0.033	0.032	0.027
20	0.051	0.040	0.035	0.038	0.037	0.031

**Table 12:** Static bedding modulus of USM no. 173 in two ranges of assessed loads.

Assessed Load Range *σ* [N/mm^2^]	Static Bedding Modulus *C*_stat_ [N/mm^3^]				
	173.1	173.2	173.3	Mean	Standard Deviation
0.009–0.035	0.0226	0.0227	0.0227	0.0227	0.0001
0.012–0.065	0.0272	0.0271	0.0272	0.0272	0.0001

**Table 13:** Static bedding modulus of USM no. 175 in two ranges of assessed loads.

Assessed Load Range *σ* [N/mm^2^]	Static Bedding Modulus *C*_stat_ [N/mm^3^]				
	175.1	175.2	175.3	Mean	Standard Deviation
0.009–0.035	0.0144	0.0131	0.0131	0.0135	0.0009
0.012–0.065	0.0162	0.0153	0.0153	0.0156	0.0006

**Table 14:** Static bedding modulus of USM no. 181 in two ranges of assessed loads.

Assessed Load Range *σ* [N/mm^2^]	Static Bedding Modulus *C*_stat_ [N/mm^3^]				
	181.1	181.2	181.3	Mean	Standard Deviation
0.009–0.035	0.0090	0.0116	0.0102	0.0103	0.0013
0.012–0.065	0.0103	0.0116	0.0113	0.0111	0.0007

## Data Availability

The original contributions presented in the study are included in the article, further inquiries can be directed to the corresponding author.
